# A network analysis of ego depletion and self-management in patients with epilepsy: differences across seizure frequencies

**DOI:** 10.3389/fpsyt.2025.1592038

**Published:** 2025-09-03

**Authors:** Xiaoxiao Yin, Jifang Cheng, Wenhao Tian, Chunjie Wen, Shengbo Jiang, Yejing Xuan, Xiuqin Feng

**Affiliations:** Department of Nursing, The Second Affiliated Hospital of Zhejiang University School of Medicine (SAHZU), Hangzhou, Zhejiang, China

**Keywords:** epilepsy, ego depletion, self-management, network analysis, seizure frequency

## Abstract

**Background:**

Self-management is essential for epilepsy control, yet many patients struggle with it, partly due to ego depletion. The interaction between ego depletion and self-management remains poorly understood in this population. This study employed network analysis to examine the interplay between ego depletion and self-management in patients with epilepsy, and to compare network structures across seizure frequency groups.

**Methods:**

A total of 655 patients with epilepsy completed validated self-report measures assessing ego depletion and self-management. Symptom-level associations were examined using network analysis, focusing on central and bridging components. Network comparison tests were conducted to assess differences across seizure frequency groups.

**Results:**

Key ego depletion symptoms such as “repeated unpleasant thoughts” and “memory difficulties” emerged as central nodes. “Urges to hit or smash things” and “uncontrollable temper” served as important bridge symptoms linking ego depletion and self-management. Among self-management dimensions, medication adherence and goal-setting were closely connected to depletion symptoms. No significant structural differences were found between patient subgroups based on seizure frequency.

**Conclusion:**

By identifying “urges to hit or smash things” and “uncontrollable temper” as central therapeutic targets, this study highlights the potential of network analysis in uncovering intervention opportunities that may be overlooked by traditional methods. Clinically, targeting these nodes through emotion regulation training could effectively disrupt the pathway to poor self-management in epilepsy patients, thereby improving both treatment adherence and overall quality of life.

## Highlights

- Network analysis explored ego depletion and self-management in patients with epilepsy.
- Medication management and goal-setting linked ego depletion symptoms to self-management.
- No significant differences in ego depletion’s impact across varying seizure frequencies.

## 1 Introduction

Epilepsy is a chronic neurological disorder characterized by recurrent, unprovoked seizures, affecting approximately 50 million people worldwide (1, 2). Beyond the occurrence of seizures, epilepsy is also associated with significant morbidity, disability, and mortality, imposing a substantial burden on individuals, families, and society as a whole (3, 4). The high prevalence of epilepsy and its severe consequences necessitate comprehensive management strategies that address both the biological and psychosocial aspects of the disease (5, 6).

While pharmacological treatments are the cornerstone of epilepsy management, effective long-term control of the condition requires active self-management by patients (7). Self-management encompasses a range of behaviors, including medication adherence, lifestyle modifications, emotional regulation, and the ability to cope with the psychological challenges posed by the disease (7, 8). However, the continuous demands of self-management often lead to significant psychological strain, which can impair patients’ ability to maintain these critical behaviors (9, 10). In order to adapt to both internal and external pressures, individuals must regulate various stressors, a process that demands substantial psychological resources (11). This process, known as self-regulation, involves the active management of emotional and cognitive demands (10), Baumeister’s energy model of self-control posits that self-regulation consumes psychological energy or resources, which are finite over time (12). Chronic or sustained high levels of self-control result in the depletion of these resources, leading to what is referred to as self-regulatory fatigue (13). This phenomenon, termed ego depletion, is characterized by a gradual reduction in energy and motivation resulting from prolonged emotional and cognitive stress (14, 15). Ego depletion can adversely affect self-management behaviors in patients with chronic disease, further complicating disease control and exacerbating overall health outcomes (16). Prolonged cognitive and emotional exertion depletes the mental resources needed for effective self-regulation, ultimately leading to a decline in both psychological well-being and the ability to adhere to treatment protocols (9, 17). Therefore, addressing ego depletion is essential for improving both the psychological and behavioral outcomes in patients with epilepsy.

Although numerous studies have provided empirical evidence on the association between ego depletion and self-management behaviors in chronic patients, most studies have relied on aggregate scores (18–20). Both ego depletion and self-management behaviors are multidimensional constructs typically assessed through multiple items. However, the use of aggregate scores may obscure the unique contributions of specific ego depletion symptoms and different aspects of self-management, thereby limiting mechanistic insights into their co-occurrence. A shift from overall composite scores to the level of individual items or dimensions can yield more nuanced understanding of the interplay between ego depletion and self-management behaviors (21). Furthermore, in the case of epilepsy patients, the frequency of seizures may itself influence the degree of ego depletion and self-management behaviors (22–24). However, few studies have directly compared the effects of different seizure frequencies on patients’ self-regulation and self-management.

To address these limitations, the present study adopts a novel approach using network analysis. Network analysis, which employs advanced statistical methods to model relationships between multiple variables, offers a promising approach for investigating these complex interactions (25). By employing Gaussian graphical models, network analysis provides an unbiased visualization of how different psychological and behavioral factors are interconnected, without relying on predefined assumptions about their relationships. The strength of this method lies in its ability to uncover hidden patterns of association and identify key variables that play pivotal roles in linking distinct symptom and behavior modules (25). Specifically, the bridge centrality index in network analysis can identify variables that serve as critical connectors between different communities of symptoms or behaviors, offering insight into their relative importance within the overall system (26).

Therefore, the network approach offers a novel descriptive framework for describing the interrelations between ego depletion and self-management behaviors in patients with epilepsy. The primary objectives of this study are: 1) To describe the network structure of ego depletion in relation to self-management behaviors in patients with epilepsy. 2) To describe key network characteristics based on centrality-related metrics. The secondary objective is: 3) To describe differences in overall network characteristics among patient groups with varying seizure frequencies.

## 2 Methods

### 2.1 Design and participants

#### 2.1.1 Design

This cross-sectional descriptive study was conducted from June 1^st^ to November 30^th^, 2023, at a specialist epilepsy clinic and the epilepsy center ward of a tertiary general hospital in Hangzhou, Zhejiang Province, China. The inclusion criteria were: (1) adults aged 18 years or older, (2) a confirmed diagnosis of epilepsy within the past six months, based on the International League Against Epilepsy classification system (27), as documented in medical records and verified by treating physicians, (3) the ability to read and communicate in Chinese, and (4) the absence of psychiatric disorders requiring active treatment. Exclusion Criteria: (1) Patients with intellectual impairment, (2) Patients diagnosed with severe medical conditions, cognitive impairment, or mental illnesses, (3) Incomplete data.

The sample size was determined based on network analysis guidelines. Epskamp et al. suggested that 500 participants are sufficient for partial correlation network analysis using Gaussian Graphical Models (28). For networks with 20 nodes, sparse networks require 200~500 participants, moderately dense networks require 550, and dense networks require 600 (29). Eligible patients attending the clinic during the study period were invited to participate using a convenience sampling method. Ethical approval was granted by the hospital’s institutional review board, and informed consent was obtained from all participants.

#### 2.1.2 Data collection

Data collection employed a hybrid approach, integrating both online and offline survey methods. The online survey was administered via Questionnaire Star, a widely recognized professional survey platform in China. Prior to accessing the questionnaire, Participants provided informed consent by clicking the “Next Page” button. To minimize incomplete responses, participants were allowed to pause and resume the survey at any time, with their progress automatically saved. For participants without access to a smartphone, a paper-based version of the questionnaire was provided. In addition, we offered iPads on site to facilitate online participation for those who preferred not to use paper forms. For participants who experienced difficulty completing the electronic survey, research staff were readily available nearby to provide assistance as needed. To ensure data quality and identify inattentive responses, five strategically embedded attention-check questions were included in the survey. For instance, one question explicitly instructed participants to “select the third option.” Responses failing these checks were deemed invalid. A total of 655 valid responses were received for analysis. These participants, who met the study’s eligibility criteria, were recruited using a convenience sampling method.

### 2.2 Clinical assessments

The general information questionnaire was developed by the researchers to align with the study’s objectives and content. It primarily collects demographic information (e.g., age, gender, marital status, and education level) and disease-related details (e.g., type of epilepsy, age of onset, duration of the disease, and number of medications used).

Seizure frequency was assessed through self-reports of seizure activity over the past year, with an open-ended question allowing patients to describe their frequency in their own words. Based on seizure frequency benchmarks (30), patients were classified into two groups: the high frequency group, which included patients reporting daily, weekly, or monthly seizures, or seizures occurring every few months; and the low frequency group, which included patients reporting yearly seizures, seizures every few years, or no seizures in the past year.

#### 2.2.1 Self-Regulating Fatigue Scale

The scale was developed by Australian scholar Nes et al. (31) and adapted to Chinese by Wang et al. (32).The Chinese version exhibits good reliability and validity (Cronbach’s α =  0.842) (32). It comprises 16 items designed to measure self-regulatory fatigue across three dimensions: cognition (6 items), emotion (5 items), and behavior (5 items). Responses are recorded using a 5-point Likert scale, ranging from 1 (“strongly disagree”) to 5 (“strongly agree”), yielding a total score between 16 and 80. Higher scores reflect a greater degree of self-regulatory fatigue. The scale demonstrates strong internal consistency, with a Cronbach’s α coefficient of 0.840 for the original version and 0.863 in the present study, confirming its reliability in measuring self-regulatory fatigue within this context.

#### 2.2.2 The Epilepsy Self-Management Scale (ESMS)

The ESMS, developed by Dilorio et al. (33), is a validated instrument designed to assess self-management strategies in individuals with epilepsy. The Chinese version comprises 34 items categorized into five dimensions: medication management (10 items), information management (5 items), safety management (7 items), seizure management (6 items), and lifestyle management (6 items) (34). Each item is rated on a 5-point Likert scale, ranging from 1 (“never”) to 5 (“always”), with total scores ranging from 34 to 170. Higher scores reflect better self-management abilities. The Chinese version demonstrated good internal consistency, with a reported Cronbach’s α of 0.848 (34). In the current study, the ESMS exhibited strong internal consistency, with a Cronbach’s alpha of 0.819, affirming its reliability in evaluating self-management behaviors among epilepsy patients.

## 3 Statistical analyses

The data were analyzed using R Studio version 4.4.2 (2024-10-31). Descriptive analyses were conducted using the describeBy function from the psych package (35), which reported means and standard deviations (SD) for continuous variables, and frequencies and percentages for categorical variables. Comparisons of ego depletion and self-management across different seizure frequency subgroups were performed using independent *t*-tests or one-way ANOVA, with *P* < 0.05 considered statistically significant.

### 3.1 Network estimation

The network model were conducted using R Studio version 4.4.2 (2024-10-31) within the bootnet (36) and qgraph (37) packages. The Graphical Gaussian Model (GGM) was employed in combination with the graphical least absolute shrinkage and selection operator (LASSO) and the Extended Bayesian Information Criterion (EBIC) to shrink minor edges to zero (28). To ensure accurate correlation estimation, the corMethod parameter was set to “cor_auto”, enabling the function to automatically select the most appropriate correlation method based on the data type (38).

In the network model, each item was represented as a “node”, while the relationships between items are represented as “edges” (39). The presence of an edge between two nodes signifies a relationship between the corresponding items, conditioned on all other nodes in the network. Thicker edges indicated stronger associations, while the color of the edge reflected the direction of the correlations (e.g., green edges represent positive correlations, and red edges represent negative correlations).

Nodes with higher correlations were positioned closer to one another in the figure, whereas nodes with more connections to other items tended to be located near the center. Conversely, nodes positioned further from the center indicated fewer associations. For interpretative clarity, nodes were grouped into predefined categories, such as “Cognitive Control,” “Emotional Control”, “Behavioral Control”, and “Self-Management”. These nodes were determined based on the dimensions of the ESMS.

### 3.2 Network accuracy

Bootstrap methods were employed to evaluate the precision of the network estimates. Specifically, 1000 bootstrap samples were generated to calculate confidence intervals for various network statistics, including edge weights and centrality measures (strength, closeness, betweenness, and expected influence). The bootstrapped 95% confidence intervals (CIs) and bootstrapped mean edge weights were calculated. To enhance computational efficiency, parallel computation using five cores was implemented. The bootnet function from the bootnet package was utilized for this purpose, ensuring robust estimation of the network parameters.

### 3.3 Network stability

To assess the robustness of the centrality measures and edge weights, case-dropping bootstrap analysis was conducted. This method systematically removes a proportion of observations (cases) to evaluate the stability of key network metrics, including strength and expected influence. A total of 500 bootstrap samples were generated, and parallel computation using five cores was implemented to improve computational efficiency.

The correlation stability (CS) coefficient was calculated to quantify the extent to which centrality indices remain stable under case-dropping conditions (38). The CS coefficient represents the maximum proportion of observations that can be dropped while maintaining a strong correlation (r ≥ 0.7) with the original centrality values. A CS coefficient greater than 0.25 is considered acceptable, while values above 0.5 indicate excellent stability (38).

### 3.4 Network comparison

To examine differences in network structure between seizure frequency groups, the Network Comparison Test (NCT) was conducted using the NetworkComparisonTest package in R Studio. A total of 10,000 permutation samples were generated to assess invariance measures, including edge weights, centrality indices, and bridge centrality. Statistical significance for differences between the two networks was determined using a threshold of *p* < 0.05. Centrality metrics with a stability coefficient below 0.25 were excluded from further group comparisons to ensure robust and reliable results.

## 4 Results

### 4.1 Sample characteristics

The average age of participants ranges from 18 to 73 years, with 42.40% being female. The majority of surveyed epilepsy patients are employed, live in urban areas, are single, have no family history of epilepsy, experience generalized seizures, and have not experienced seizures in the past year. The patient information for the high frequency and low frequency groups is shown in **Table 1**, with further details provided in **Supplementary Table S1**. **Table 2** presents the means and standard deviations for all items.

**Table 1:** Demographic and clinical characteristics of epilepsy patients by seizure frequency.

Category	Mean (standard deviation) / composition ratio (%)		P-value
	High frequency group	Low frequency group	
Gender			1
Male	148 (51.93)	229 (61.89)	
Female	137 (48.07)	141 (38.11)	
Age(years)			0.004
Mean ± SD	33.23 ± 12.98	30.45 ± 11.01	
Location			1
Urban	134 (47.02)	226 (61.08)	
Rural	151 (52.98)	144 (38.92)	
Marital status			0.213
Unmarried	150 (52.63)	211 (57.03)	
Married	117 (41.05)	154 (41.62)	
Divorced	14 (4.91)	4 (1.08)	
Widowed	4 (1.4)	1 (0.27)	
Education level			0.224
Elementary school and below	34 (11.93)	8 (2.16)	
Junior high school	83 (29.12)	59 (15.95)	
High school or vocational school	55 (19.3)	57 (15.41)	
Associate degree	58 (20.35)	105 (28.38)	
Bachelor’s degree	52 (18.25)	123 (33.24)	
Graduate degree	3 (1.05)	18 (4.86)	
Employment status			0.242
Student	36 (12.63)	74 (20)	
Underemployed	32 (11.23)	21 (5.68)	
Employed	113 (39.65)	213 (57.57)	
Homemaker	36 (12.63	16 (4.32)	
Unemployed	52 (18.25)	34 (9.19)	
Retired	16 (5.61)	12 (3.24)	
Average household income (in RMB/month)			0.220
≤2000	43 (15.09)	18 (4.86)	
2000–5000 (including 5000)	110 (38.6)	93 (25.14)	
5000–10000 (including 10000)	79 (27.72)	131 (35.41)	
10000–20000 (including 20000)	34 (11.93)	68 (18.38)	
>20000	19 (6.67)	60 (16.22)	
Major type of epilepsy			0.220
Focal aware seizures	50 (17.54)	50 (13.51)	
Focal impaired awareness seizures	47 (16.49)	39 (10.54)	
Generalized seizures	86 (30.18)	131 (35.41)	
Unclear/not classifiable	36 (12.63)	53 (14.32)	
Unknown	66 (23.16)	97 (26.22)	

**Table 2:** Description, mean, and SD for network nodes.

	Variable	High frequency group		Low frequency group		t-statistic	P-value
		Mean	SD	Mean	SD		
Cognition	ego1	2.835	0.818	2.961	0.811	−1.969	0.049
	ego2	2.765	0.831	2.982	0.882	−3.210	0.001
	ego3	3.068	0.851	3.214	0.852	−2.183	0.029
	ego5	2.776	0.875	2.895	0.886	−1.715	0.087
	ego11	2.803	0.884	3.018	0.966	−2.927	0.004
	ego13	3.011	0.931	3.189	0.919	−2.453	0.014
Emotion	ego4	2.868	0.972	3.126	0.956	−3.410	0.001
	ego6	2.995	0.971	3.228	0.916	−3.151	0.002
	ego8	3.046	0.949	3.281	0.918	−3.197	0.001
	ego10	3.222	0.855	3.446	0.801	−3.444	0.001
	ego16	2.749	0.973	2.902	0.970	−2.000	0.046
Behavior	ego7	3.416	0.775	3.709	0.674	−5.158	0.000
	ego9	3.170	0.837	3.368	0.852	−2.974	0.003
	ego12	3.276	0.836	3.537	0.753	−4.195	0.000
	ego14	3.308	0.915	3.561	0.848	−3.662	0.000
	ego15	3.173	0.903	3.484	0.842	−4.545	0.000
Self-management behaviors	SELF1	45.149	5.378	43.723	5.786	3.223	0.001
	SELF2	10.973	4.530	9.821	4.198	3.363	0.001
	SELF3	24.959	3.794	23.874	3.803	3.626	0.000
	SELF4	24.016	4.378	23.172	4.423	2.433	0.015
	SELF5	19.938	5.594	18.751	5.830	2.629	0.009

### 4.2 Network structure

**Figure 1:**
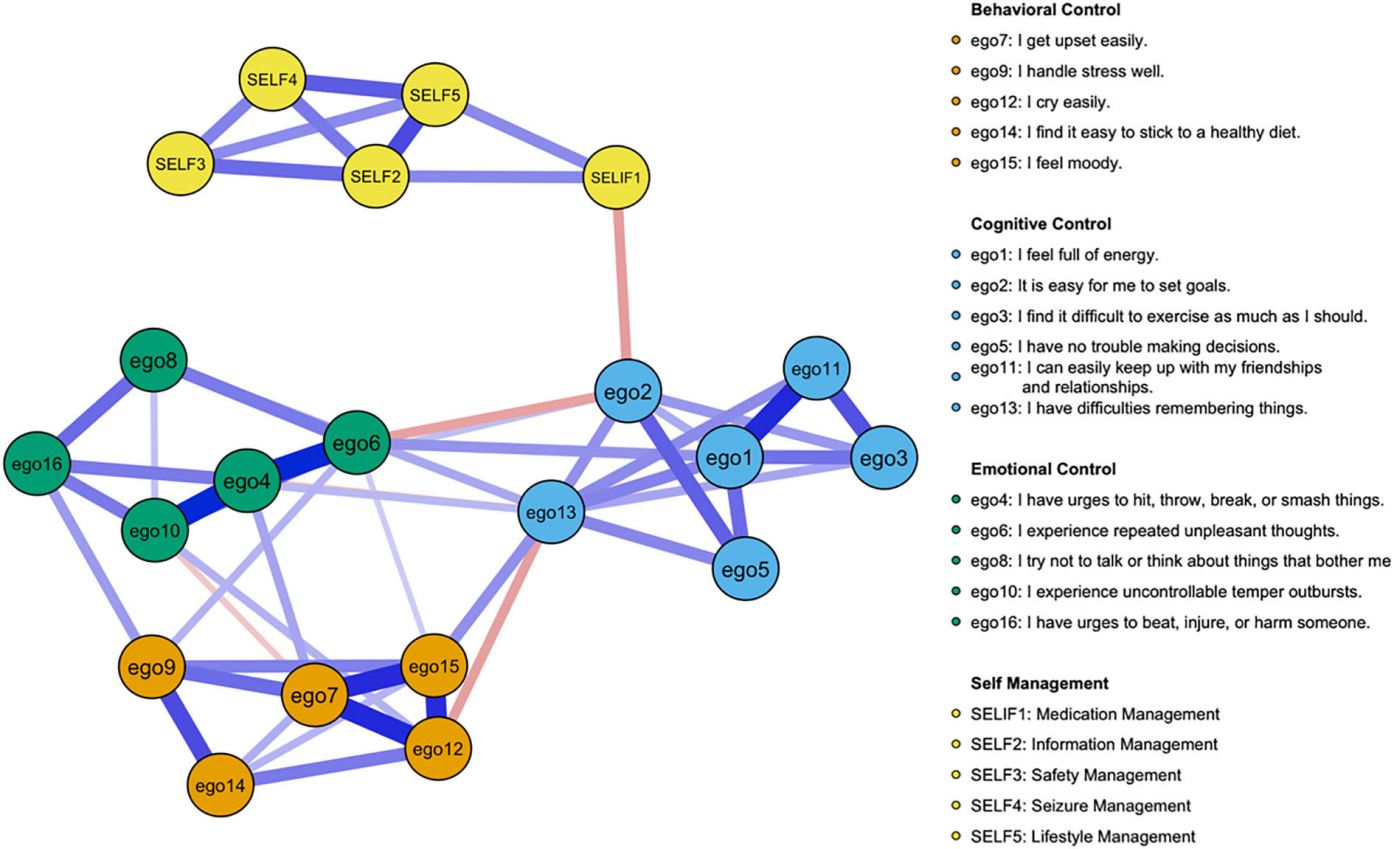
Network structure of ego depletion symptoms and self-management behaviors. Green edges represent positive correlations, red edges represent negative correlations. The thickness of the edge reflects the magnitude of the correlation. (To enhance clarity, the following changes were made to the variable names: ego1 becomes Energy, ego2 becomes Goal Setting, ego3 becomes Exercise Difficulty, ego5 becomes Decision Making, ego11 becomes Social Relationships, ego13 becomes Memory Issues, ego4 becomes Aggression, ego6 becomes Negative Thoughts, ego8 becomes Avoidance, ego10 becomes Temper Outbursts, ego16 becomes Violence Urges, ego7 becomes Upset Easily, ego9 becomes Stress Handling, ego12 becomes Cry Easily, ego14 becomes Diet Adherence, ego15 becomes Mood Swings, SELF1 becomes Medication Management, SELF2 becomes Information Management, SELF3 becomes Safety Management, SELF4 becomes Seizure Management, and SELF5 becomes Lifestyle Management).

The network model is presented in **Figure 1** (see **Supplementary Table S2** for more information on the correlation matrix). The entire network comprises a total of 52 edges, with edge weights ranging from −0.092 to 0.364. Notably, only one edge connects the ego depletion community to the self-management behaviors community.

**Figure 2:**
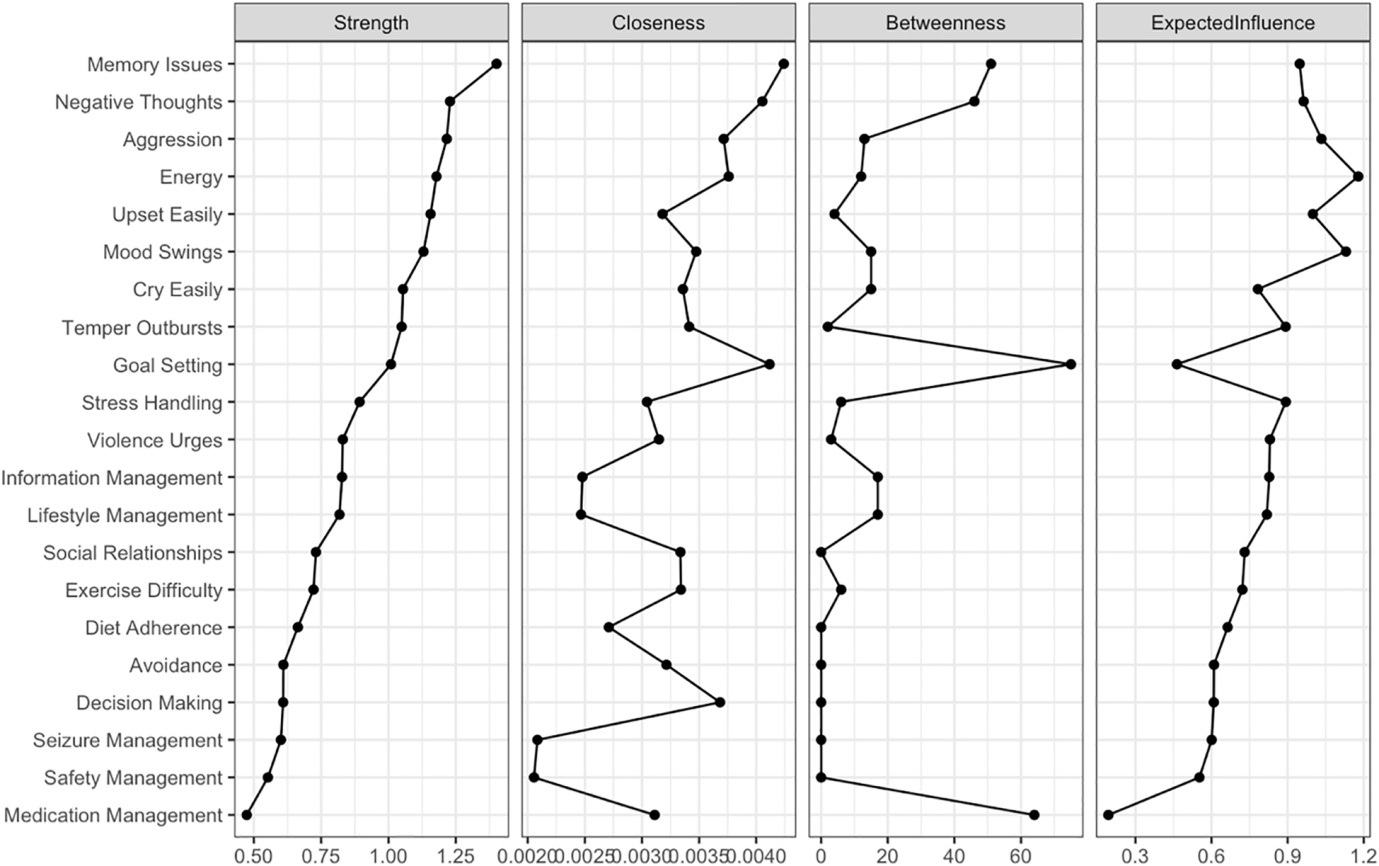
Centrality indices of the network model.

**Figure 3:**
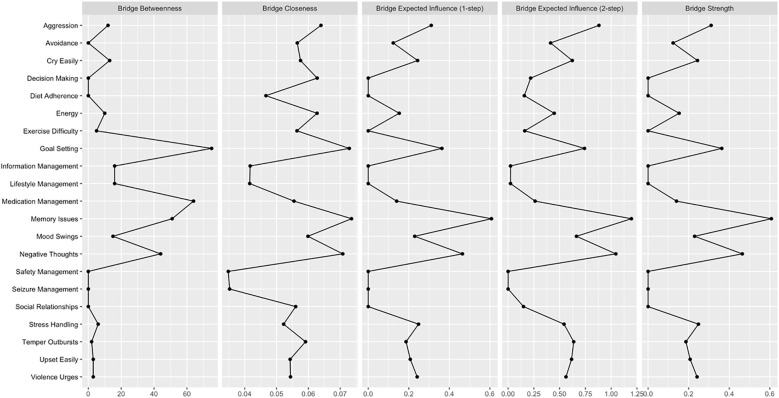
Bridge centrality indices of the network model.

**Figure 2** displays the node centrality indices (see **Supplementary Table S3** Centrality Indices). Among all nodes, “Memory Issues” exhibits the highest node strength (strength = 1.402), indicating its substantial direct connectivity with other nodes. This is followed closely by “Negative Thoughts” (strength = 1.229). In contrast, “Medication Management” demonstrates the lowest node strength (strength = 0.473) in the network. The bridge centrality results are illustrated in **Figure 3**. A detailed examination of the network (refer to **Figure 1**) reveals that the four identified communities form relatively stable internal structures while maintaining notable interconnections. Notably, the most robust edge is “Medication Management” – “Goal Setting” (weight = −0.140) in the network connects the self-management behaviors community to the general ego depletion community.

### 4.3 Network stability and accuracy

In the network of Ego Depletion and Self-Management symptoms, the 95% confidence intervals of edge weights are narrow, suggesting that the estimates of these weights are precise and reliable (**Supplementary Figure S1**). As demonstrated in **Supplementary Figure S2**, the average correlations of strength and expected influence (EI) indices between the original sample and subsamples decrease steadily as subsample size reduces, reflecting the natural loss of stability with smaller samples. In this context, the CS coefficient for strength reaches 0.362, exceeding the threshold of 0.25, which indicates acceptable stability. In comparison, the CS coefficient for EI achieves 0.516, surpassing the more rigorous threshold of 0.5, indicating a high level of stability for EI. Significance tests for strength differences identify “Memory Issues” as the most central and influential node, exhibiting significantly greater strength compared to all other nodes (**Supplementary Figure S3**). This finding underscores the dominant role of “Memory Issues” in connecting and stabilizing the network. Additionally, the analysis of edge weight differences reveals that the edge weight between “Aggression” and “Temper Outbursts” is the largest among all significant edges, emphasizing its critical importance in shaping the overall network structure (**Supplementary Figure S4**).

### 4.4 Network comparison

**Figure 4:**
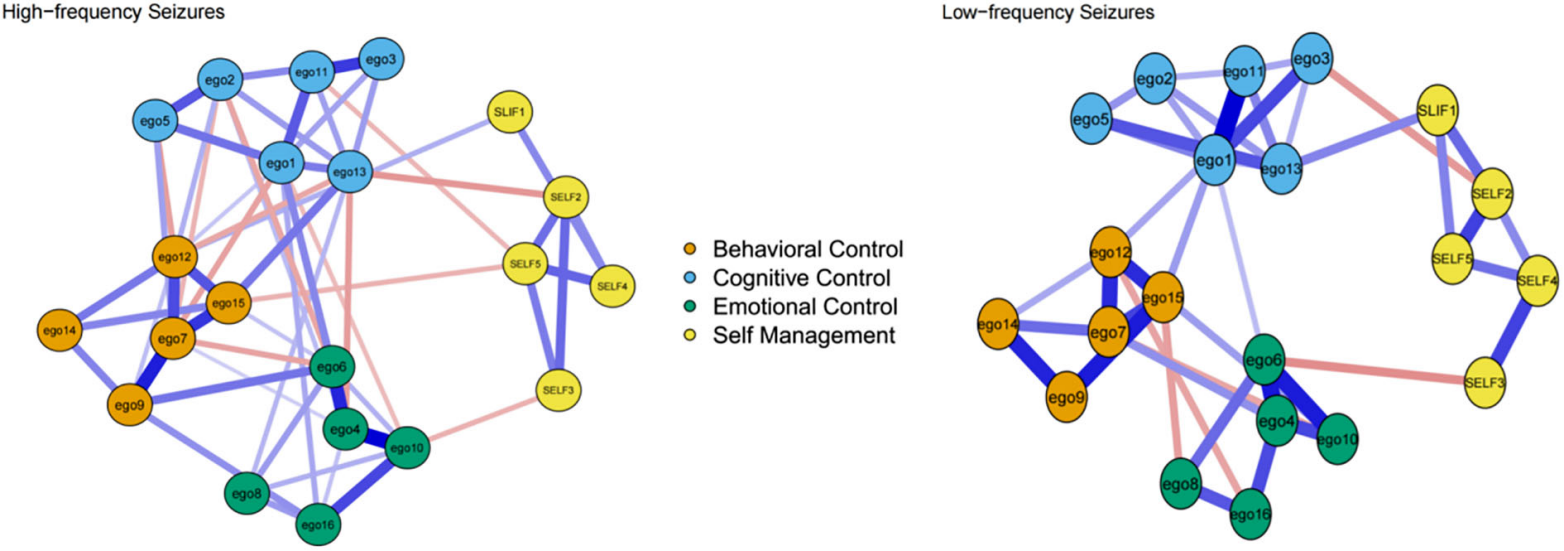
Network structure of the combined model for the high-frequency and low-frequency seizure groups.

The combined network models for the two seizure frequency groups are shown in **Figure 4**. A comparison of the two networks indicates no significant differences in global network strength (**Supplementary Figure S5**), with the high-frequency group exhibiting a value of 9.608 and the low-frequency group exhibiting 9.641 (S = 0.033, p = 0.814). Additionally, the distribution of edge weights between the two groups does not differ significantly (M = 0.202, p = 0.135). Additionally, it is important to note that the only statistically significant difference between the two groups was in age (p = 0.004).

## 5 Discussion

This study investigates the relationship between ego depletion symptoms and self-management behaviors in patients with epilepsy using network analysis. To our knowledge, this is the first study to apply network analysis to explore this relationship, offering new insights into how ego depletion interacts with and influences self-management behaviors. Among the total sample of 655 patients, 370 participants (56.41%) reported high seizure frequency (daily, weekly, monthly, or every few months), while 285 participants (43.59%) experienced low seizure frequency (yearly, every few years, or no seizures in the past year).

Within the components of ego depletion symptoms, the present study identifies the strongest connection between “Aggression” and “Temper Outbursts”. This relationship highlights the close link between impulsive aggression and uncontrolled emotional responses in patients with epilepsy. This finding aligns with theoretical frameworks suggesting that emotional dysregulation frequently manifests as physical impulses and temper loss, underscoring the interconnectedness of these symptoms within the domain of emotional control (40, 41). The other three strongest edges are between “Aggression” and “Negative Thoughts”, between “Energy” and “Social Relationships”, between “Upset Easily” and “Stress Handling”. These relationships highlight the interplay between intrusive thoughts and impulsive behavior, the role of energy in social functioning, and the link between emotional sensitivity and stress management. Together, these results deepen our understanding of the interconnections among ego depletion symptoms, particularly within the realms of emotional and cognitive control. By identifying these critical relationships, the study highlights potential intervention targets, such as addressing repetitive negative thoughts, enhancing energy levels to improve social functioning, and strengthening stress management skills to mitigate emotional reactivity.

In the ego depletion network, “Negative Thoughts” and “Memory Issues” demonstrate the highest overall centrality, indicating that these two variables play a critical role within the current network comprising 16 ego depletion symptoms and 5 epilepsy self-management behaviors. These findings suggest that “Negative Thoughts” and “Memory Issues” may serve as effective breakthrough points for reducing ego depletion in epilepsy patients. Cognitive issues, such as memory difficulties, can hinder medication adherence and the identification of seizure triggers, which may in turn increase seizure frequency (42). Simultaneously, repeated unpleasant thoughts can exacerbate emotional distress, impair decision-making abilities, and weaken motivation for self-management (43). This bidirectional relationship between ego depletion and poor self-management behaviors further aggravates cognitive and emotional difficulties, creating a cycle that lowers patients’ overall quality of life (44, 45). Addressing these central symptoms through targeted interventions, such as cognitive training to improve memory (46) and strategies to manage intrusive thoughts (47), may help break this cycle.

In the network structure of self-management behaviors, all items are tightly interconnected, forming a stable and cohesive system. The present study identifies the strongest connection between “Information Management” and “Lifestyle Management”, a finding with significant implications. This robust association suggests that effective information management is closely linked to lifestyle management behaviors. These behaviors appear to mutually reinforce one another, indicating that improved access to and utilization of health information can directly support healthier lifestyle choices in patients with epilepsy (48). Moreover, “Information Management” exhibits the highest centrality within the self-management behaviors network, underscoring its pivotal role in driving and sustaining the overall structure. As the central node, information management serves as a key facilitator for other self-management behaviors. Patients who effectively acquire, process, and apply relevant health information are more likely to engage in essential practices, such as medication adherence, safety precautions, and lifestyle adjustments (22, 49, 50). By strengthening this critical component, patients may achieve better self-regulation, reduce seizure frequency, and enhance their overall quality of life.

The network analysis shows that connections between ego depletion symptoms and self-management behaviors are sparse, with only one significant, strongly weighted, and negatively directed edge identified. This finding aligns with the theory of ego depletion (9, 15), which posits that cognitive and emotional fatigue weakens self-control, impairing the ability to perform resource-intensive tasks such as lifestyle management and information processing. However, the absence of additional significant associations suggests that the negative effects of ego depletion may only become evident under specific conditions or for behaviors that demand substantial cognitive or emotional resources (51). Consistent with dual-system theory (52–54), self-management behaviors – particularly routine tasks such as medication adherence or maintaining a structured lifestyle – tend to become automated over time, reducing their reliance on active self-regulation. As a result, even when patients with epilepsy experience ego depletion, they can still effectively execute these critical behaviors because such actions have been internalized as habitual routines or are reinforced through external cues and environmental support (54). This mechanistic shift lessens the dependence on conscious self-regulation, enabling patients to sustain critical self-management activities despite cognitive and emotional fatigue. This result aligns with Perez’s findings (55), which indicate that ego depletion has a diminished impact on behaviors that are automated or habitual, as these require minimal active self-regulatory effort.

Although the high-frequency seizure group demonstrates lower scores for ego depletion symptoms, the network structure reveals a greater number of edges, indicating more extensive interconnections among symptoms. These connections, while relatively weaker, are more prevalent and widely distributed within the high-frequency group. In contrast, the low-frequency seizure group exhibits higher ego depletion symptom scores, yet the network structure contains fewer edges with stronger connections. The lack of significant differences in statistical results can be primarily attributed to this distinction: the high-frequency group exhibits a greater quantity of edges with weaker weights, whereas the low-frequency group shows fewer edges with stronger weights (56). This observation highlights a noteworthy phenomenon: despite experiencing milder symptoms, the high-frequency group’s broader and more fragmented interconnections among symptoms may increase the overall complexity and challenges of self-management. The dispersed and widespread nature of these symptom interactions likely imposes greater demands on patients’ cognitive and emotional resources, complicating their ability to prioritize tasks, allocate limited self-regulatory efforts effectively, and maintain efficient condition management (9).

## 6 Implications for clinical practice

This study provides important insights into the link between ego depletion and self-management behaviors in patients with epilepsy. Healthcare providers should pay particular attention to those with high seizure frequency, who are more prone to cognitive and emotional fatigue. Practical interventions may include cognitive-behavioral therapy to reduce repetitive negative thinking, and mindfulness-based techniques to improve emotional regulation. For patients experiencing memory or attention difficulties, cognitive training and planning tools can support better medication adherence and seizure tracking. To minimize the impact of ego depletion on behavior, clinicians are encouraged to promote habit formation through digital tools such as reminder apps or structured self-monitoring routines (57, 58). In addition, psychoeducational and peer-support programs tailored to seizure frequency may enhance emotional resilience and provide social reinforcement for self-management practices. Routine assessment of fatigue levels using brief screening tools during follow-up visits can help adjust interventions in a timely manner. These strategies highlight the importance of personalized, sustainable care that supports both psychological functioning and daily self-management in people with epilepsy.

## 7 Limitations

This study has several limitations that should be acknowledged. First, data were collected through self-reported questionnaires, which may be subject to recall bias and social desirability bias. Second, the use of a convenience sample drawn from a single tertiary hospital in China may significantly limit the generalizability of the findings. Cultural norms, health literacy, and differences in healthcare systems may influence self-management behaviors in ways that are not captured in this setting. Therefore, caution should be exercised when applying these results to other regions or populations. Third, the network analysis in this study estimated between-subject effects at the group level, and it is important to recognize that network properties, such as centrality and structure, may not remain consistent at the individual level. Finally, the study did not incorporate a longitudinal design, preventing the exploration of dynamic changes in network structure over time.

## 8 Conclusion

This study explores the complex relationships between symptoms of ego depletion and self-management behaviors in patients with epilepsy. The results identify repeated unpleasant thoughts and memory difficulties as the most central symptoms, highlighting their significant role in the overall network. Additionally, impulsive aggression and uncontrollable temper outbursts are key symptoms that connect different symptom groups. Medication management and goal-setting were also found to be important links between symptoms and behaviors. These findings suggest that interventions targeting the most central symptoms could help reduce ego depletion and improve self-management behaviors. Specifically, strategies to manage intrusive thoughts, improve memory, regulate emotions, and enhance medication adherence and goal-setting skills may be particularly beneficial. Such targeted approaches could improve self-management and, ultimately, the quality of life for individuals with epilepsy. This highlights the value of personalized, network-based interventions in clinical practice.

## Data Availability

The raw data supporting the conclusions of this article will be made available by the authors, without undue reservation.
