# Supplementary material for: A network analysis of ego depletion and self-management in patients with epilepsy: differences across seizure frequencies
Source: Front Psychiatry. 2025 Sep 3;16:1592038. doi: 10.3389/fpsyt.2025.1592038 (PMC12441827; doi:10.3389/fpsyt.2025.1592038)
Supplement: Supplementary file 1 [file Supplementaryfile1.docx]

**Table S1** Patients’ demographic characteristics and epilepsy-related clinical features

| Category | Mean (Standard Deviation) / Composition Ratio (%) | | | | | | | |
| --- | --- | --- | --- | --- | --- | --- | --- | --- |
|  | Total | High frequency group | | | | Low frequency group | | |
|  |  | Daily seizures | Weekly seizures | Monthly seizures | Seizures every few months | Yearly seizures | Seizures every few years | No seizures in the past year |
| **Gender** |  |  |  |  |  |  |  |  |
| Male | 377 (57.60) | 18 (47.37) | 19 (40.43) | 48 (48.48) | 63 (62.38) | 43 (55.13) | 26 (70.27) | 160 (62.75) |
| Female | 278(42.40) | 20 (52.63) | 28 (59.57) | 51 (51.52) | 38 (37.62) | 35 (44.87) | 11 (29.73) | 95 (37.25) |
| **Age（years）** |  |  |  |  |  |  |  |  |
| Mean ± SD | 31.66 ±11.98 | 28.26 ± 9.66 | 35.09 ± 14.81 | 33.52 ± 12.89 | 33.95 ± 12.99 | 32.77 ± 13.11 | 31.41 ± 12.21 | 29.61 ± 10.02 |
| **Location** |  |  |  |  |  |  |  |  |
| Urban | 360(55.00) | 9 (23.68) | 24 (51.06) | 49 (49.49) | 52 (51.49) | 44 (56.41) | 26 (70.27) | 156 (61.18) |
| Rural | 295(45.00) | 29 (76.32) | 23 (48.94) | 50 (50.51) | 49 (48.51) | 34 (43.59) | 11 (29.73) | 99 (38.82) |
| **Marital status** |  |  |  |  |  |  |  |  |
| Unmarried | 361(55.11) | 22 (57.89) | 26 (55.32) | 50 (50.51) | 52 (51.49) | 40 (51.28) | 21 (56.76) | 150 (58.82) |
| Married | 271(41.38) | 14 (36.84) | 19 (40.43) | 40 (40.4) | 44 (43.56) | 38 (48.72) | 15 (40.54) | 101 (39.61) |
| Divorced | 18(2.75) | 2 (5.26) | 1 (2.13) | 6 (6.06) | 5 (4.95) | 0 (0) | 1 (2.7) | 3 (1.18) |
| Widowed | 5(0.76) | 0 (0) | 1 (2.13) | 3 (3.03) | 0 (0) | 0 (0) | 0 (0) | 1 (0.39) |
| **Education level** |  |  |  |  |  |  |  |  |
| Elementary school and below | 42(6.41) | 3 (7.89) | 5 (10.64) | 8 (8.08) | 18 (17.82) | 1 (1.28) | 0 (0) | 7 (2.75) |
| Junior high school | 142(21.68) | 14 (36.84) | 15 (31.91) | 27 (27.27) | 27 (26.73) | 11 (14.1) | 4 (10.81) | 44 (17.25) |
| High school or vocational school | 112(17.10) | 9 (23.68) | 16 (34.04) | 19 (19.19) | 11 (10.89) | 19 (24.36) | 2 (5.41) | 36 (14.12) |
| Associate degree | 163(24.89) | 9 (23.68) | 7 (14.89) | 21 (21.21) | 21 (20.79) | 23 (29.49) | 15 (40.54) | 67 (26.27) |
| Bachelor’s degree | 175(26.72) | 3 (7.89) | 4 (8.51) | 21 (21.21) | 24 (23.76) | 20 (25.64) | 16 (43.24) | 87 (34.12) |
| Graduate degree | 21(3.20) | 0 (0) | 0 (0) | 3 (3.03) | 0 (0) | 4 (5.13) | 0 (0) | 14 (5.49) |
| **Employment status** |  |  |  |  |  |  |  |  |
| Student | 110(16.79) | 5 (13.16) | 2 (4.26) | 13 (13.13) | 16 (15.84) | 12 (15.38) | 8 (21.62) | 54 (21.18) |
| Underemployed | 53(8.09) | 8 (21.05) | 10 (21.28) | 5 (5.05) | 9 (8.91) | 3 (3.85) | 2 (5.41) | 16 (6.27) |
| Employed | 326(49.77) | 12 (31.58) | 19 (40.43) | 43 (43.43) | 39 (38.61) | 50 (64.1) | 22 (59.46) | 141 (55.29) |
| Homemaker | 52(7.94) | 6 (15.79) | 5 (10.64) | 13 (13.13) | 12 (11.88) | 2 (2.56) | 1 (2.7) | 13 (5.1) |
| Unemployed | 86(13.13) | 6 (15.79) | 7 (14.89) | 19 (19.19) | 20 (19.8) | 6 (7.69) | 2 (5.41) | 26 (10.2) |
| Retired | 28(4.28) | 1 (2.63) | 4 (8.51) | 6 (6.06) | 5 (4.95) | 5 (6.41) | 2 (5.41) | 5 (1.96) |
| **Average household income (in RMB/month)** | | | | | | | | |
| ≤2000 | 61(9.31) | 2 (5.26) | 8 (17.02) | 20 (20.2) | 13 (12.87) | 8 (10.26) | 0 (0) | 10 (3.92) |
| 2000 - 5000 (including 5000 ) | 203(30.99) | 25 (65.79) | 16 (34.04) | 29 (29.29) | 40 (39.6) | 23 (29.49) | 8 (21.62) | 62 (24.31) |
| 5000 - 10000 (including 10000 ) | 210(32.06) | 9 (23.68) | 16 (34.04) | 29 (29.29) | 25 (24.75) | 22 (28.21) | 12 (32.43) | 97 (38.04) |
| 10000 - 20000 (including 20000 ) | 102(15.58) | 1 (2.63) | 5 (10.64) | 17 (17.17) | 11 (10.89) | 14 (17.95) | 12 (32.43) | 42 (16.47) |
| ＞20000 | 79(12.06) | 1 (2.63) | 2 (4.26) | 4 (4.04) | 12 (11.88) | 11 (14.1) | 5 (13.51) | 44 (17.25) |
| **Major type of epilepsy** |  |  |  |  |  |  |  |  |
| Focal aware seizures | 100(15.27) | 15 (39.47) | 8 (17.02) | 19 (19.19) | 8 (7.92) | 15 (19.23) | 1 (2.7) | 34 (13.33) |
| Focal impaired awareness seizures | 86(13.13) | 4 (10.53) | 8 (17.02) | 22 (22.22) | 13 (12.87) | 8 (10.26) | 4 (10.81) | 27 (10.59) |
| Generalized seizures | 217(33.12) | 6 (15.79) | 15 (31.91) | 28 (28.28) | 37 (36.63) | 33 (42.31) | 13 (35.14) | 85 (33.33) |
| Unclear/not classifiable | 89(13.59) | 1 (2.63) | 6 (12.77) | 14 (14.14) | 15 (14.85) | 8 (10.26) | 12 (32.43) | 33 (12.94) |
| Unknown | 163(24.89) | 12 (31.58) | 10 (21.28) | 16 (16.16) | 28 (27.72) | 14 (17.95) | 7 (18.92) | 76 (29.8) |


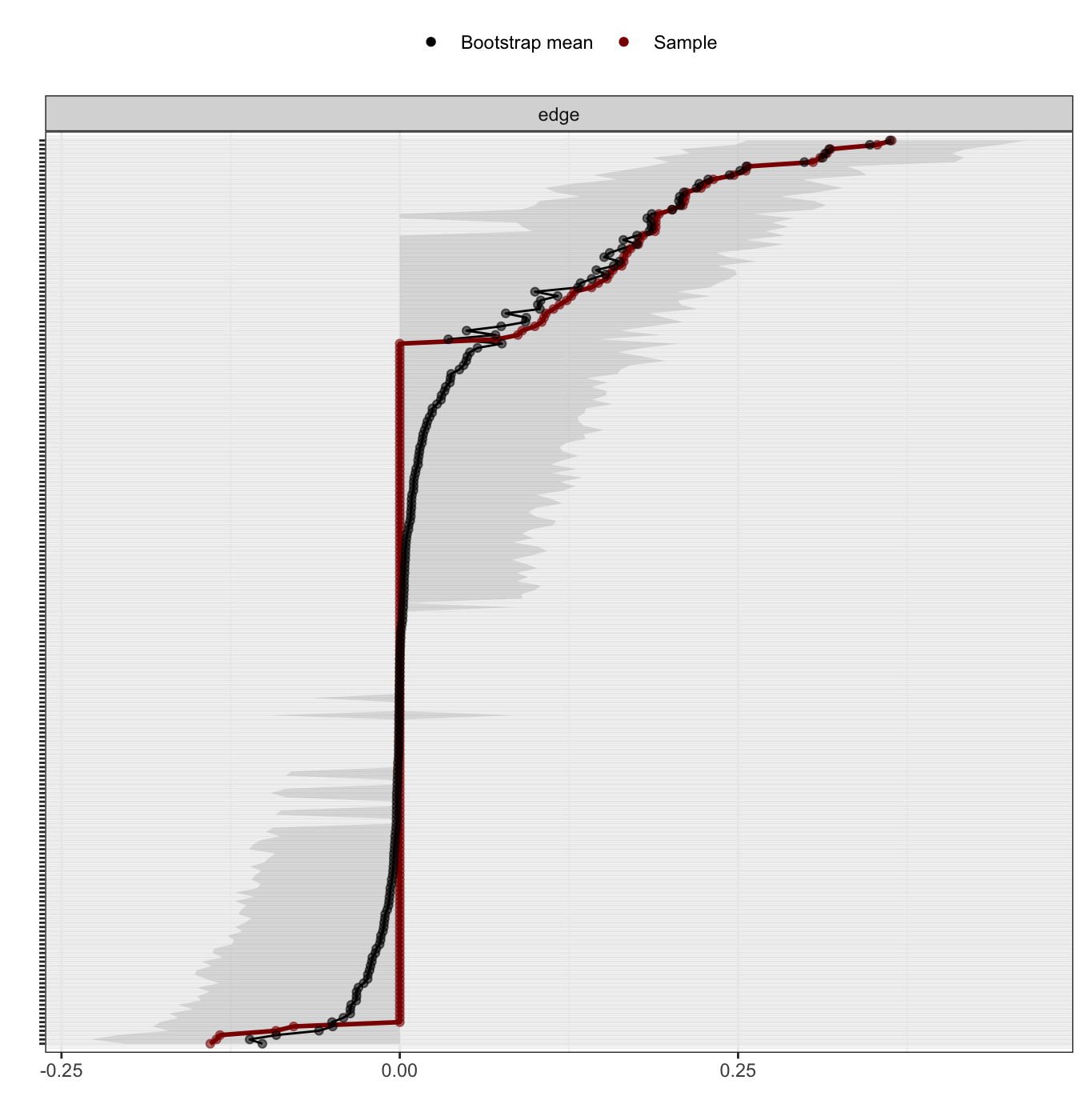


**Fig. S1** The accuracy of edge weights. The red line indicates the sample values and the gray area indicates the bootstrapped CIs. The y-axis labels have been removed to avoid cluttering.


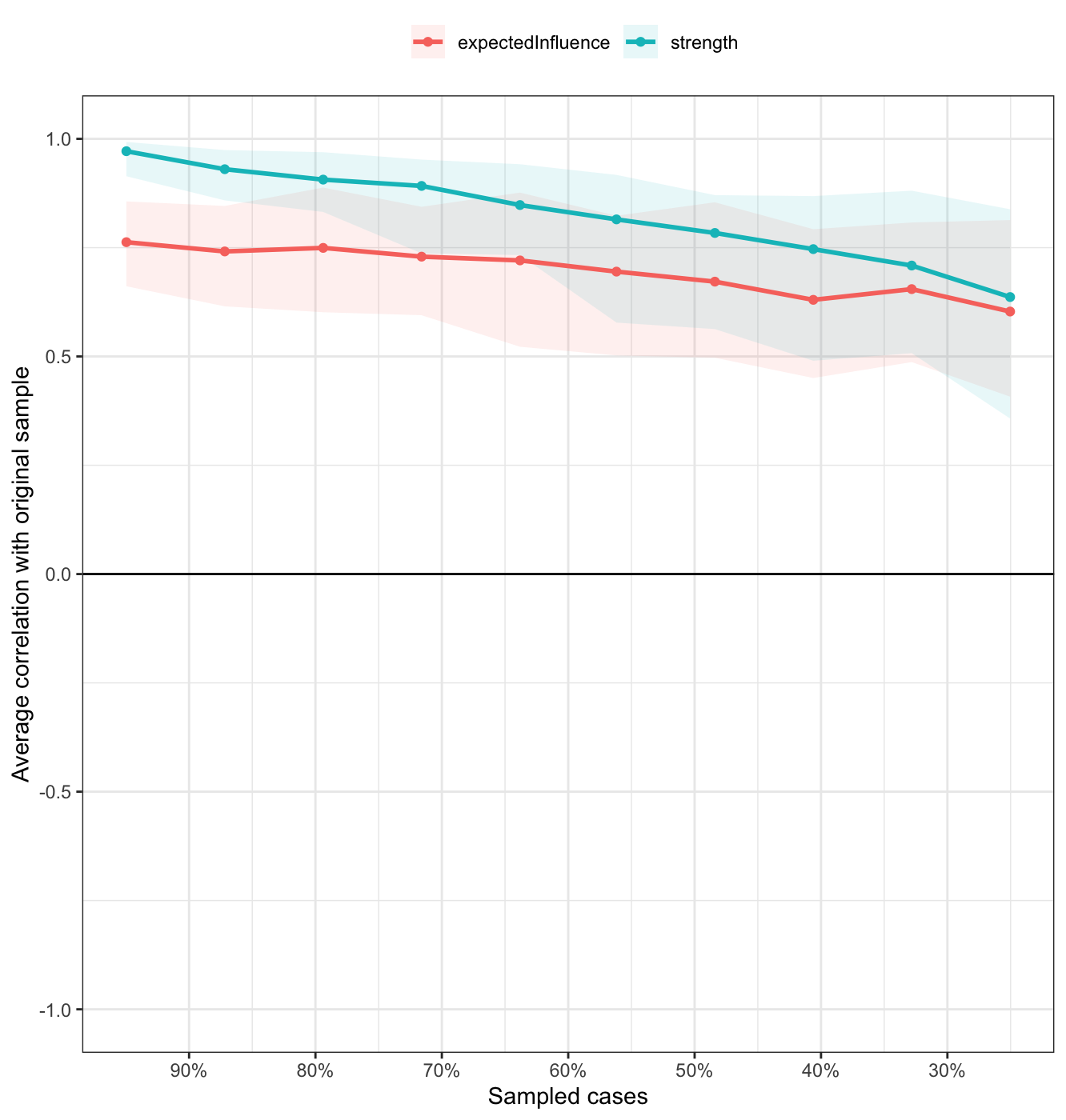
 **Fig. S2** Evaluation of the Stability of expectedInfluence centrality and strength Centrality. The red line and green line represent the average correlation between centrality indices in the full sample and subsamples. The red area and green area depict the range from the 2.5th quantile to the 97.5th quantile.


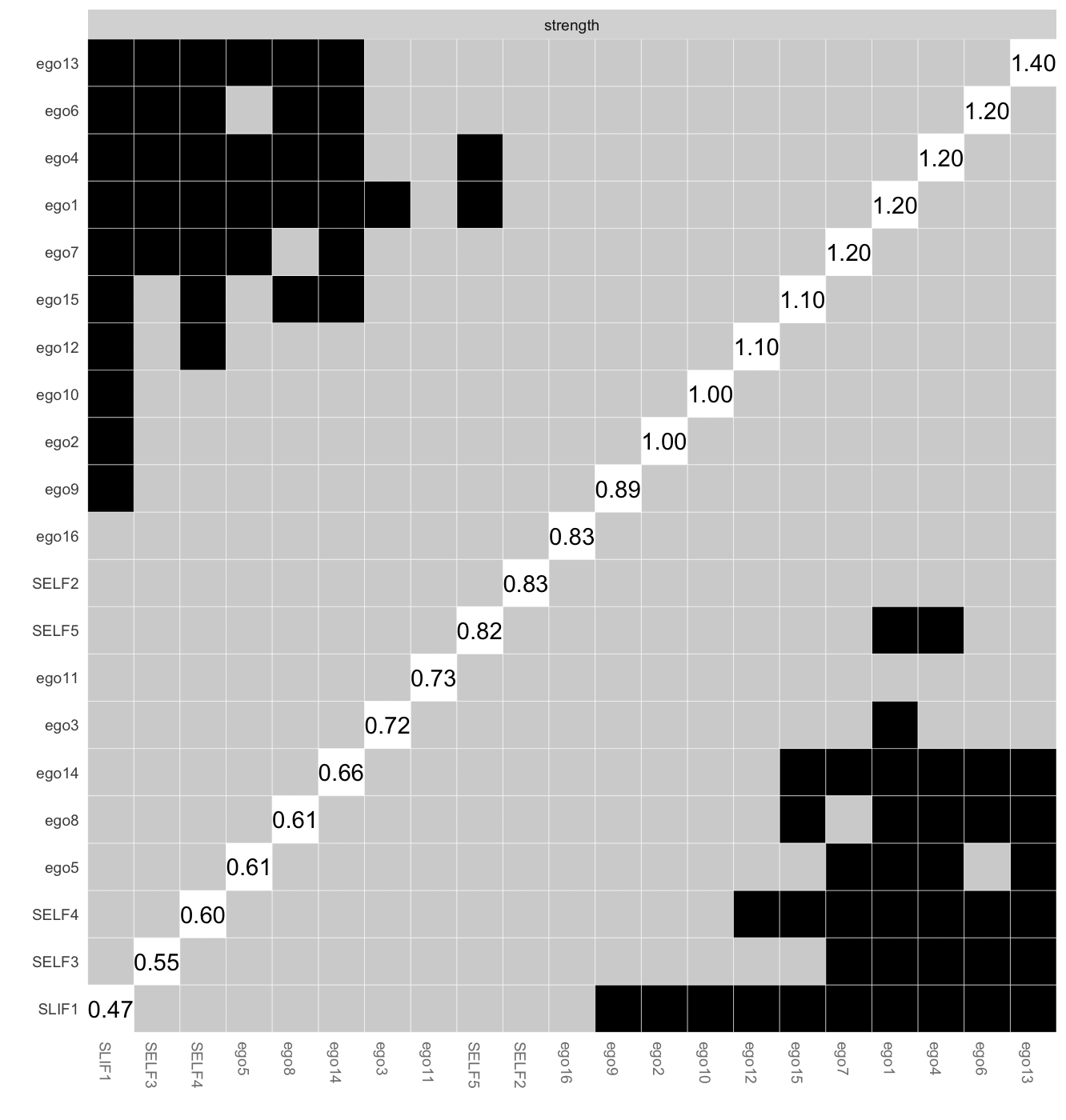
**Figure S3. Bootstrapped difference test for strength**

*Note:* Gray boxes indicate strength that do not differ significantly from one another, while black boxes indicate node strength that do differ significantly. The number in the white boxes (i.e., diagonal line) represent the value of node strength.


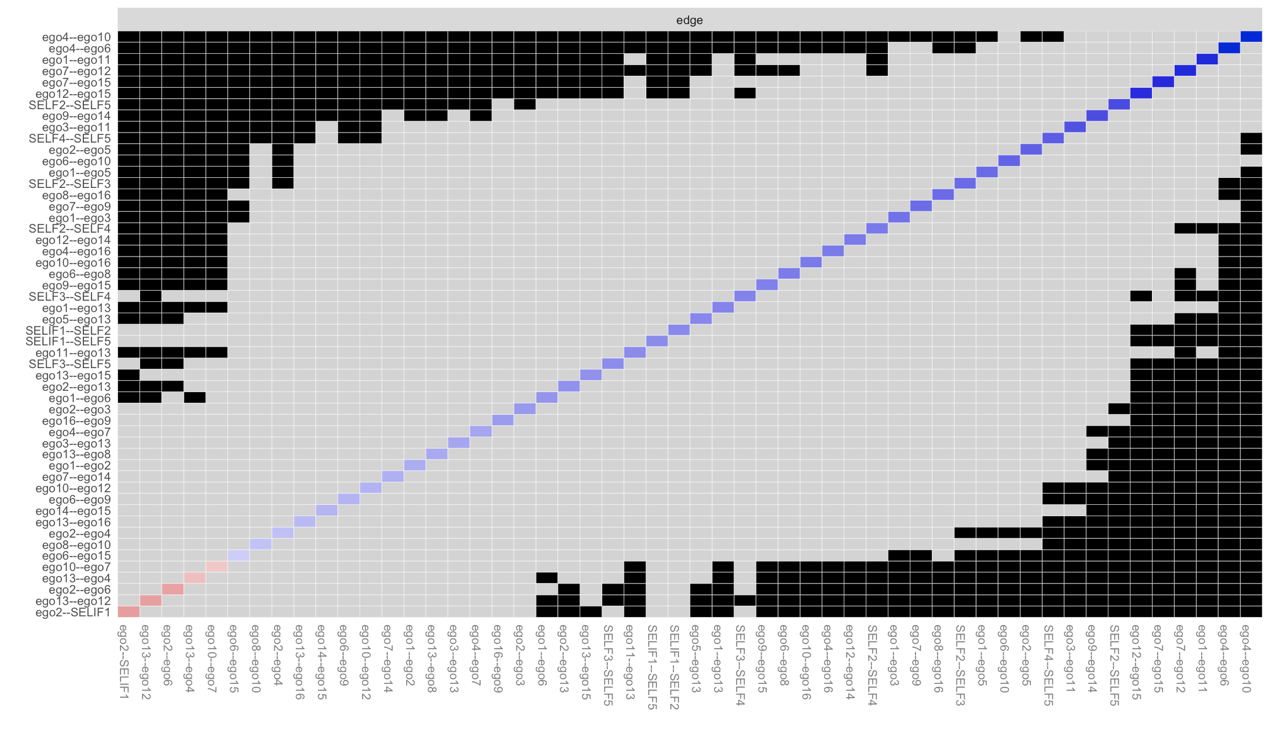


**Figure S4. Bootstrapped difference test for edge weights**

*Note:* Gray boxes indicate edge weights that do not differ significantly from one another, while black boxes indicate edge weights that do differ significantly. Blue and red boxes on the diagonal correspond to edge weights with positive and negative correlations, respectively.

**Table S2** Correlation Matrix of Ego depletion and Self-management Items

| **Item** | **ego1** | **ego2** | **ego3** | **ego5** | **ego11** | **ego13** | **ego4** | **ego6** | **ego8** | **ego10** | **ego16** | **ego7** | **ego9** | **ego12** | **ego14** | **ego15** | **SELF1** | **SELF2** | **SELF3** | **SELF4** | **SELF5** |
| --- | --- | --- | --- | --- | --- | --- | --- | --- | --- | --- | --- | --- | --- | --- | --- | --- | --- | --- | --- | --- | --- |
| **ego1** |  | 0.118 | 0.201 | 0.212 | 0.318 | 0.176 | 0.000 | 0.153 | 0.000 | 0.000 | 0.000 | 0.000 | 0.000 | 0.000 | 0.000 | 0.000 | 0.000 | 0.000 | 0.000 | 0.000 | 0.000 |
| **ego2** | 0.118 |  | 0.147 | 0.227 | 0.000 | 0.155 | 0.090 | -0.133 | 0.000 | 0.000 | 0.000 | 0.000 | 0.000 | 0.000 | 0.000 | 0.000 | -0.140 | 0.000 | 0.000 | 0.000 | 0.000 |
| **ego3** | 0.201 | 0.147 |  | 0.000 | 0.247 | 0.127 | 0.000 | 0.000 | 0.000 | 0.000 | 0.000 | 0.000 | 0.000 | 0.000 | 0.000 | 0.000 | 0.000 | 0.000 | 0.000 | 0.000 | 0.000 |
| **ego5** | 0.212 | 0.227 | 0.000 |  | 0.000 | 0.171 | 0.000 | 0.000 | 0.000 | 0.000 | 0.000 | 0.000 | 0.000 | 0.000 | 0.000 | 0.000 | 0.000 | 0.000 | 0.000 | 0.000 | 0.000 |
| **ego11** | 0.318 | 0.000 | 0.247 | 0.000 |  | 0.165 | 0.000 | 0.000 | 0.000 | 0.000 | 0.000 | 0.000 | 0.000 | 0.000 | 0.000 | 0.000 | 0.000 | 0.000 | 0.000 | 0.000 | 0.000 |
| **ego13** | 0.176 | 0.155 | 0.127 | 0.171 | 0.165 |  | -0.092 | 0.000 | 0.123 | 0.000 | 0.100 | 0.000 | 0.000 | -0.136 | 0.000 | 0.157 | 0.000 | 0.000 | 0.000 | 0.000 | 0.000 |
| **ego4** | 0.000 | 0.090 | 0.000 | 0.000 | 0.000 | -0.092 |  | 0.353 | 0.000 | 0.364 | 0.189 | 0.129 | 0.000 | 0.000 | 0.000 | 0.000 | 0.000 | 0.000 | 0.000 | 0.000 | 0.000 |
| **ego6** | 0.153 | -0.133 | 0.000 | 0.000 | 0.000 | 0.000 | 0.353 |  | 0.189 | 0.223 | 0.000 | 0.000 | 0.107 | 0.000 | 0.000 | 0.072 | 0.000 | 0.000 | 0.000 | 0.000 | 0.000 |
| **ego8** | 0.000 | 0.000 | 0.000 | 0.000 | 0.000 | 0.123 | 0.000 | 0.189 |  | 0.087 | 0.210 | 0.000 | 0.000 | 0.000 | 0.000 | 0.000 | 0.000 | 0.000 | 0.000 | 0.000 | 0.000 |
| **ego10** | 0.000 | 0.000 | 0.000 | 0.000 | 0.000 | 0.000 | 0.364 | 0.223 | 0.087 |  | 0.189 | -0.078 | 0.000 | 0.108 | 0.000 | 0.000 | 0.000 | 0.000 | 0.000 | 0.000 | 0.000 |
| **ego16** | 0.000 | 0.000 | 0.000 | 0.000 | 0.000 | 0.100 | 0.189 | 0.000 | 0.210 | 0.189 |  | 0.000 | 0.142 | 0.000 | 0.000 | 0.000 | 0.000 | 0.000 | 0.000 | 0.000 | 0.000 |
| **ego7** | 0.000 | 0.000 | 0.000 | 0.000 | 0.000 | 0.000 | 0.129 | 0.000 | 0.000 | -0.078 | 0.000 |  | 0.209 | 0.316 | 0.114 | 0.311 | 0.000 | 0.000 | 0.000 | 0.000 | 0.000 |
| **ego9** | 0.000 | 0.000 | 0.000 | 0.000 | 0.000 | 0.000 | 0.000 | 0.107 | 0.000 | 0.000 | 0.142 | 0.209 |  | 0.000 | 0.256 | 0.180 | 0.000 | 0.000 | 0.000 | 0.000 | 0.000 |
| **ego12** | 0.000 | 0.000 | 0.000 | 0.000 | 0.000 | -0.136 | 0.000 | 0.000 | 0.000 | 0.108 | 0.000 | 0.316 | 0.000 |  | 0.189 | 0.305 | 0.000 | 0.000 | 0.000 | 0.000 | 0.000 |
| **ego14** | 0.000 | 0.000 | 0.000 | 0.000 | 0.000 | 0.000 | 0.000 | 0.000 | 0.000 | 0.000 | 0.000 | 0.114 | 0.256 | 0.189 |  | 0.105 | 0.000 | 0.000 | 0.000 | 0.000 | 0.000 |
| **ego15** | 0.000 | 0.000 | 0.000 | 0.000 | 0.000 | 0.157 | 0.000 | 0.072 | 0.000 | 0.000 | 0.000 | 0.311 | 0.180 | 0.305 | 0.105 |  | 0.000 | 0.000 | 0.000 | 0.000 | 0.000 |
| **SELF1** | 0.000 | -0.140 | 0.000 | 0.000 | 0.000 | 0.000 | 0.000 | 0.000 | 0.000 | 0.000 | 0.000 | 0.000 | 0.000 | 0.000 | 0.000 | 0.000 |  | 0.168 | 0.000 | 0.000 | 0.166 |
| **SELF2** | 0.000 | 0.000 | 0.000 | 0.000 | 0.000 | 0.000 | 0.000 | 0.000 | 0.000 | 0.000 | 0.000 | 0.000 | 0.000 | 0.000 | 0.000 | 0.000 | 0.168 |  | 0.211 | 0.192 | 0.257 |
| **SELF3** | 0.000 | 0.000 | 0.000 | 0.000 | 0.000 | 0.000 | 0.000 | 0.000 | 0.000 | 0.000 | 0.000 | 0.000 | 0.000 | 0.000 | 0.000 | 0.000 | 0.000 | 0.211 |  | 0.177 | 0.164 |
| **SELF4** | 0.000 | 0.000 | 0.000 | 0.000 | 0.000 | 0.000 | 0.000 | 0.000 | 0.000 | 0.000 | 0.000 | 0.000 | 0.000 | 0.000 | 0.000 | 0.000 | 0.000 | 0.192 | 0.177 |  | 0.232 |
| **SELF5** | 0.000 | 0.000 | 0.000 | 0.000 | 0.000 | 0.000 | 0.000 | 0.000 | 0.000 | 0.000 | 0.000 | 0.000 | 0.000 | 0.000 | 0.000 | 0.000 | 0.166 | 0.257 | 0.164 | 0.232 |  |

**Table S3** Centrality Indices

| Item | Betweenness | Closeness | Strength | ExpectedInfluence |
| --- | --- | --- | --- | --- |
| ego1 | 12 | 0.004 | 1.178 | 1.178 |
| ego2 | 75 | 0.004 | 1.009 | 0.464 |
| ego3 | 6 | 0.003 | 0.721 | 0.721 |
| ego5 | 0 | 0.004 | 0.609 | 0.609 |
| ego11 | 0 | 0.003 | 0.731 | 0.731 |
| ego13 | 51 | 0.004 | 1.402 | 0.947 |
| ego4 | 13 | 0.004 | 1.216 | 1.033 |
| ego6 | 46 | 0.004 | 1.229 | 0.963 |
| ego8 | 0 | 0.003 | 0.610 | 0.610 |
| ego10 | 2 | 0.003 | 1.049 | 0.892 |
| ego16 | 3 | 0.003 | 0.830 | 0.830 |
| ego7 | 4 | 0.003 | 1.156 | 0.999 |
| ego9 | 6 | 0.003 | 0.893 | 0.893 |
| ego12 | 15 | 0.003 | 1.054 | 0.783 |
| ego14 | 0 | 0.003 | 0.663 | 0.663 |
| ego15 | 15 | 0.003 | 1.130 | 1.130 |
| SELF1 | 64 | 0.003 | 0.473 | 0.193 |
| SELF2 | 17 | 0.002 | 0.827 | 0.827 |
| SELF3 | 0 | 0.002 | 0.552 | 0.552 |
| SELF4 | 0 | 0.002 | 0.600 | 0.600 |
| SELF5 | 17 | 0.002 | 0.818 | 0.818 |

**
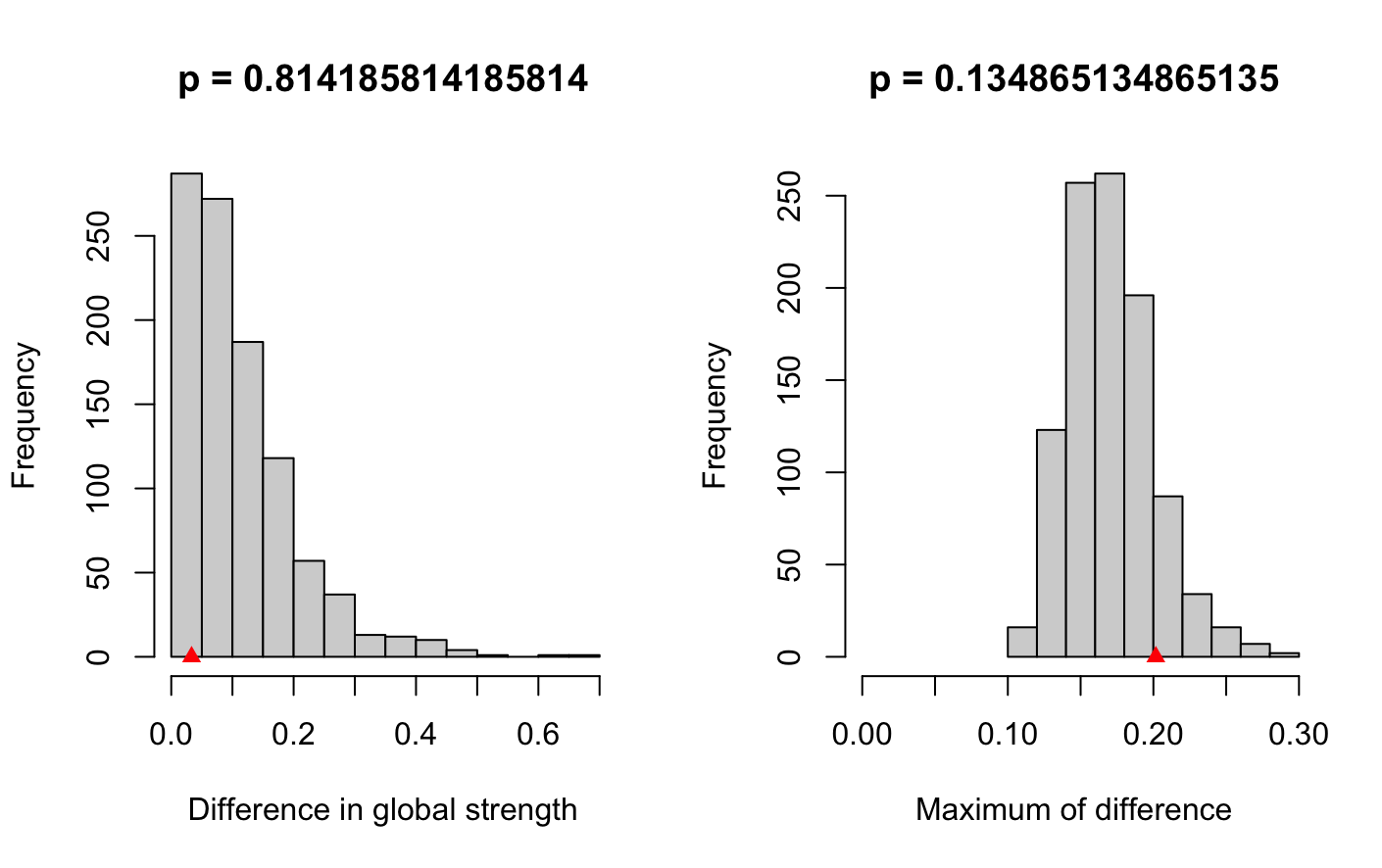
**

**Fig.S5** Comparison of Network Properties Between the High-Frequency and Low-Frequency Seizure Groups. The left panel displays the bootstrap analysis of differences in global network strength, showing no significant difference between the two groups (high-frequency group: 9.608; low-frequency group: 9.641; S = 0.033, p = 0.814). The right panel illustrates the bootstrap results for the maximum difference in edge weights, based on 1,000 permutations, which also reveals no significant difference (M = 0.202, p = 0.135).
